# Improving the Robustness of Odour Recognition with Odour-Image Data Fusion in Open-Air Settings

**DOI:** 10.3390/s26082493

**Published:** 2026-04-17

**Authors:** Fanny Monori, Alin Tisan

**Affiliations:** Department of Electronic Engineering, Royal Holloway, University of London, Egham TW20 0EX, UK

**Keywords:** odour recognition, image classification, multimodality, decision fusion, gas sensors, fruit ripening

## Abstract

Odour recognition with low-cost gas sensors is challenging in open-air settings due to the non-specificity of the sensors and environmental variability. This can be mitigated by incorporating additional information into the classification process. This paper investigates odour-image multimodality in two case-studies of increasing complexity: banana ripening in open-air environment and strawberry ripening in a glasshouse environment. Data were collected using custom acquisition platforms equipped with cameras and MOX gas sensors operated with temperature modulation. For the visual modality, image classification (MobileNetV3) and object detection (YoloV5) models are trained. For the odour modality, established classical machine learning methods (Random Forest, XGBoost, SVM and Logistic Regression) and neural networks (1D-CNN, LSTM, MLP, and ELM) are employed. Each modality is analysed independently and together to critically assess scenarios in which combining modalities provides a clear advantage over using either modality alone. Results show that models trained on odour data achieve high accuracy in controlled environments but underperform in more dynamic open-air settings. Image-based models are sensitive to the image quality in all environments; however, they are more robust when deployed in different environments. Lastly, it is demonstrated that decision fusion consistently increases the accuracy, by as much as +12.36% in the banana ripening and +3.63% in the strawberry ripening scenario. Where decision fusion does not improve classification accuracy significantly, it is shown that the multimodal approach can still be leveraged to identify high-confidence predictions by selecting samples where both modalities agree on the label.

## 1. Introduction

It has been demonstrated in various application fields [1,2] that combining multiple types of data (modalities) into one classification system increases the performance of the whole system. This increase in performance has been stated to be measurable in gains [3,4,5]. Gains can be achieved in accuracy (the accuracy of the classification), certainty (the belief in the correctness of the predictions), completeness (the increase in the overall information) and representation (the abstractness of the system). Multimodal data fusion has been explored in application areas such as healthcare [6], computer vision [7] and robotics [8]. However, it remains relatively under-explored in odour recognition, in a field where the aim is to automatically recognise an object by its smell.

Odour recognition can be performed with an array of low-cost metal-oxide (MOX) gas sensors, by the algorithmic processing of the response signals of the sensors. Such a system is often referred to as the electronic nose (EN). However, these sensors have low specificity [9] and they are susceptible to sensor drift [10]. Concentration of the target gas, sensors’ position relative to the gas source [11], and wind [12] are additional factors that influence the quality of the signal. Because of these challenges, it is preferred to do odour recognition in a clean laboratory environment and in closed-sampling systems, so that these influencing factors can be controlled. Other analytical technologies exist that are more precise and robust, such as gas chromatography-mass spectrometry (GC-MS) and infrared (IR) spectroscopy, but they are expensive and laborious and limited to laboratory environments [13,14]. Sensors with better specificity and better robustness against drift exist, such as IR-source gas sensors [13,15], but they are larger and more expensive than MOX sensors, and not suitable for small and low-cost portable EN systems.

As the ultimate goal is to deploy EN systems in dynamic or “real-world” environments for automated monitoring, these systems should ideally be low-cost, portable, and capable of dealing with the challenges of non-specificity and sensor drift. Introducing additional information (another modality) into the task can help mitigate the problems of MOX gas sensors. However, it is imperative to determine the possible gains when adopting a multimodal approach. Introducing another data source increases the cost and the complexity of the system, therefore it should be used only in scenarios where a significant amount of gain can be expected. Applications in controlled environments may already perform well with only one modality. In contract, in dynamic, real-world environments, where both modalities may suffer from significant limitations, combining multiple types of data may provide a better view of the problem.

Such application where multimodality may increase the performance of the system exists in food monitoring and precision agriculture scenarios. Fruit ripening is such a scenario, where both the appearance of the fruits as well as its smell are correlated with the ripening process. A strawberry visibly gets more red as it ripens, and a banana turns from green to yellow to brown throughout its ripening process. The visual appearance of fruits is generally the primary basis for ripeness assessment in commercial settings. This assessment is most often done manually by workers or in laboratories using analytical equipment [16], with the aim of adhering to established marketing standards and guidelines [17,18,19]. However, this manual assessment is laborious and slow, therefore automating the process is desirable. Computer-vision based automatic fruit ripeness classification methods are well-explored in the literature [20,21,22,23,24,25]. Similarly, ripening causes a change in the chemical profile of a fruit, which has been evaluated as the basis of ripeness classification via odour recognition [26,27,28,29,30].

On the other hand, both odour and image modality in these scenarios have several drawbacks. Odour data is affected by low sensor specificity and sensor drift, and is susceptible to environmental variability. The consequences of these physical limitations of MOX gas sensors are expected to be severe in real-life fruit ripening scenarios, such as in a commercial glass-house, a poly-tunnel or on an open strawberry field. Image modality faces other challenges, all of which are difficult to mitigate at all times. Fruit occlusion by leaves hinders computer vision based detection [31,32], while variations in lighting can degrade the image quality [22,23]. In addition, both the proximity of the camera to the fruit [33] and out-of-focus images [34] have also been reported to be a problem.

We hypothesise that a multimodal camera and gas sensor based recognition approach under such challenging conditions may provide a better system, and it can be measured by an increase in either the accuracy, the confidence, or the completeness of the system. Combining image and gas sensor data has been proposed by other works in different contexts, which are summarised in Table 1. These works can be grouped into two categories, based on the aim of the modality fusion. Most works that combined gas sensor data with image modalities did so to achieve a greater understanding of the chemical process that is being investigated [35,36,37,38,39,40,41,42,43]. These works typically use a commercial portable EN for the odour sensing, and hyper-spectral cameras or a combination of hyper-spectral and RGB cameras for the visual modality. However, hyper-spectral imaging is expensive, data acquisition with available commercial electronic noses is laborious and not automated, therefore this technology, while well-suited for precise measurements in laboratory environments, may not translate well into low-cost automated monitoring systems.

Another line of work is concerned with building and evaluating multimodal systems that can be used in real-life application scenarios. This second group of works is closest to the aim of this paper. Similarly to this work, Chen et al. [28] investigated multimodal fusion with banana ripening in a closed-sampling scenario. Kim et al. [44] proposed an image and gas sensor based IoT platform for meat quality monitoring, also in a closed-sampling setting. Narkhede et al. [45] combined image data with open-air gas sensor data to classify perfume and smoke fumes. However, these studies were conducted in controlled laboratory or near-laboratory environments. Consequently, it cannot be assumed that data fusion will improve performance in all settings, as in real-world environments both modalities may suffer from significant limitations. In a controlled environment Chen et al. [28] already achieved high accuracy with individual modalities (>98%), leaving limited opportunity for improvement with a multimodal approach (which increased the accuracy to 100%). It is similarly difficult to assess the true benefit of data fusion in Kim et al. [44], who did not report the performance of the modalities separately, potentially overestimating the advantage of data fusion (with which they achieved 99.44%) in a closed-sampling scenario. Finally, the open-air setting used by Narkhede et al. [45] is more similar to deployed applications; however, they took images only of the gas plume instead of a specific object, which is not suitable for fruit ripening or other agricultural monitoring applications. Their fusion results (96%) also provided only modest improvement over the unimodal accuracy (93%).

To address these research gaps, this work investigates whether multimodal fusion can provide measurable benefits under real-world conditions with low-cost equipment, in settings where it is expected that one or both modalities may face limitations. We investigated multimodality in two fruit ripening scenarios with increasing complexity. One scenario investigates the classification of banana ripening in a controlled but open-air environment, and a second case-study investigates the classification of strawberry ripening in a glass-house. Both of these case-studies were developed to examine the research gap in a dynamic setting, and to also determine whether a low-cost gas-sensor and camera set-up is viable in potential commercial settings. For both of these case-studies data collection was carried out. For the odour modality, MOX gas sensors were used with temperature modulation type data acquisition in an open-air setup, while image data was acquired using simple low-cost RGB cameras in an automated manner. Odour and image data were evaluated separately with classical machine learning models and neural networks. The models and modalities were also evaluated with decision fusion, following maximum confidence and majority voting strategies. Both the unimodal and the multimodal results are presented compared.

To the best of our knowledge, this is the first study that critically evaluates the advantage of using odour-image multimodal data fusion through different scenarios, and the first to combine open-air temperature-modulated odour acquisition with image data. This work is also the first to investigate strawberry ripening under such conditions, and the first to combine image and odour data for this task.

## 2. Materials

### 2.1. Data Collection on Fruit Ripening

The aim of this paper was to critically analyse the advantage of multimodal approaches in odour recognition scenarios compared to unimodal approaches, and for that data was collected for two fruit ripening scenarios in open sampling settings: banana ripening and strawberry ripening. For the data collection, custom data acquisition platforms were assembled following a temperature modulation approach for the gas sensors, and the platforms and data acquisition protocols are detailed in this section.

#### 2.1.1. Data Acquisition Equipment

Three EN equipment were assembled with 5 MOX tin-oxide (${\mathrm{SnO}}_{2}$) gas sensors from Figaro Engineering Inc. (Osaka, Japan), TGS2612, TGS2611, TGS2602, TGS2600 and TGS2620 (Table 2), and a humidity and temperature sensor. The sensors were chosen based on their sensitivity to compounds that have been shown to correlate with fruit ripening (specifically strawberry) maturity, such as VOCs, ethanol, organic solvents, alcohol, methane [26,27]. These sensors provide complementary, but slightly overlapping sensitivity to the target analytes.

**Figure 1:**
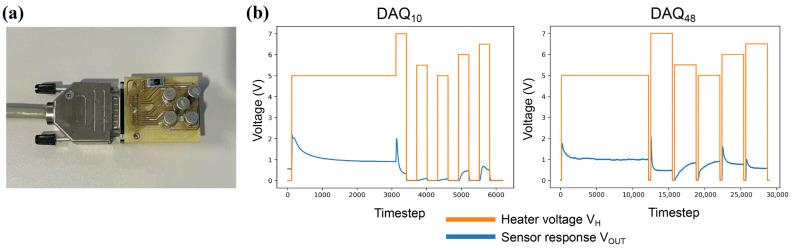
(**a**) The sensor board used for the data acquisition. (**b**) Temperature modulation patterns ${\mathrm{DAQ}}_{10}$ and ${\mathrm{DAQ}}_{48}$.

Each of the three sensor boards (shown on Figure 1a) with the gas sensors were connected to a NI MyRio 1900 board (National Instruments, Austin, TX, USA), which was running the data acquisition. The MyRio boards were configured to run with a 10 MHz sampling rate, and the data was saved onto USB drives. Furthermore, a Raspberry Pi 4b (Raspberry Pi Ltd., Cambridge, UK) was also used in the data acquisition set up separately for the image acquisition. Two cameras were used in the set-up, a low-light wide-angle 1080p60 machine vision camera (See3CAM_CU30 from e-con Systems, Fremont, CA, USA) and an indoor HD web camera.

#### 2.1.2. Odour Data Acquisition Strategies

For two of the acquisition boards, the odour acquisition was configured to run with a temperature modulation approach to induce the transitorial behaviour (response curve) of gas sensors reacting to target analytes. Two configurations of voltage modulation patterns were applied onto the heater element of the gas sensors ($V_{H}$), a longer and a shorter acquisition pattern, which are illustrated in Table 3 and Figure 1b. This work followed a similar pattern to the one defined in [46]. Both patterns started with a pre-heating period (5 min and 20 min), followed by different alternating voltage levels for 5 and 28 min. Having alternating voltage levels provides a more comprehensive set of features for analysis compared to a single voltage-level approach. The shorter acquisition length was configured to run for a total of 10 min (6000 data points), while the longer acquisition ran for a total of 48 min (28,800 data points). These two acquisition platforms will be referred to as ${\mathrm{DAQ}}_{10}$ and ${\mathrm{DAQ}}_{48}$ for the rest of this paper. A third configuration was also tested for one of the data collection scenarios, where the sensors were continuously applied 5 V to $V_{H}$, denoted as ${\mathrm{DAQ}}_{cont}$.

These three approaches were selected based on the following considerations. ${\mathrm{DAQ}}_{48}$ was expected to provide more stable and stronger features, as there is more time for the sensors to get saturated. However, for real-life applications, shorter acquisition time is preferable, thus ${\mathrm{DAQ}}_{10}$ was also evaluated. On the other hand, both temperature modulation strategies induce the transient sensor response by operating above the recommended heater voltage ($V_{H}$), which is 5 V. Therefore, a third approach was also included where the sensors were heated continuously in line with the specifications set by the manufacturer. However, this requires a different data processing strategy, as the resulting data lacks the characteristic response-curve shape.

#### 2.1.3. Case-Study 1: Banana Ripening in an Open-Air Setting

**Figure 2:**
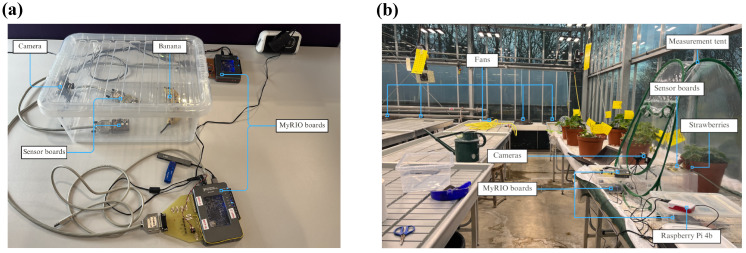
The set-up for the data collection case-studies. (**a**) Case study 1: observing banana ripening. (**b**) Case-study 2: strawberry ripening inside a glasshouse.

The first case-study focused on banana ripening classification, assigning ripe or overripe labels to the fruits. This data collection was run inside an office environment, and while temperature and humidity remained largely stable, background human activity and the unenclosed setup made this scenario potentially challenging. At any given time, one ripe or overripe banana was placed in a sampling set-up, and were alternated every few days. Data were collected over several weeks, between 21 November 2024 and 15 December 2024, to form the training dataset and the first hold-out test set (batch 1 test data). A second batch of data was acquired one month later, between 9 January 2025 and 5 February 2025. Overall, 400 training and 88 and 461 test samples were acquired with ${\mathrm{DAQ}}_{10}$. See Table 4 for a summary of the dataset. The longer acquisition (${\mathrm{DAQ}}_{48}$) configuration was also set-up, however, exploratory data analysis showed similar characteristics to ${\mathrm{DAQ}}_{10}$, therefore this data was excluded from further analysis to avoid redundancy. The set-up for this data acquisition is shown on Figure 2a. Note that the container’s lid was removed for the data collection.

The labelling of the data was based on the visual appearance of the bananas, and the following strategy was used. Bananas that were predominantly or entirely brown were labelled as “overripe” samples, whereas bananas that were either uniformly yellow or had brown spots were labelled as “ripe” bananas. Furthermore, when acquiring data for batch 1, the sensor boards were placed in close proximity to the fruit, with a variable distance of approximately 1–5 cm. For batch 2, the sensor boards were positioned consistently such that they were in direct contact with the fruit. This data-collection approach allowed comparison between data reflecting realistic usage (batch 1) and data optimised for repeatability (batch 2). In potential open-air deployment scenarios, the object being measured may not always be positioned at a consistent distance, and algorithms should be robust to such variability. However, it must be noted, that due to the one month pause between collecting batch 1 and 2, sensor drift acts as a confounding factor, with prior studies reporting that such drift can affect the data as early as one week [47] or several weeks [10] after deployment.

#### 2.1.4. Case-Study 2: Strawberry Ripening in a Glasshouse

A second case-study was designed to evaluate the odour-image multimodal approach in a scenario that is closer to a real-life fruit monitoring application. For that, ripening of strawberries in a glasshouse environment was chosen to be studied. This was motivated by the research effort to evaluate low-cost technologies for optimal ripening time prediction of soft fruits [48]. In this work, this was simplified to a classification problem, where strawberry plants were classified based on whether they have ripe fruits or not, creating the two target labels: “ripe” and “unripe”.

**Figure 3:**
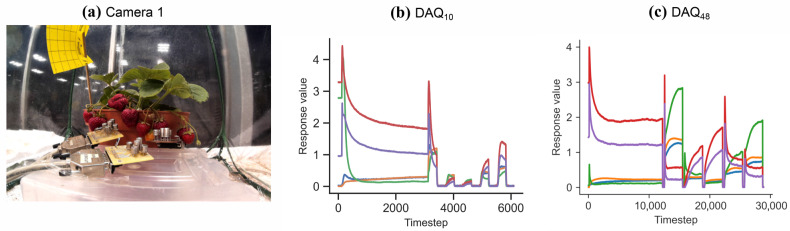
Example data taken from strawberry dataset. (**a**) An example image from the See3CAM_CU30 camera. (**b**) An odour data sample acquired with ${\mathrm{DAQ}}_{10}$. (**c**) An odour data sample acquired with ${\mathrm{DAQ}}_{48}$.

12 *Fragaria x ananassa* (strawberry) seedlings were purchased, and the plants were placed inside the temperature controlled research glasshouse of Royal Holloway, University of London. The strawberries were placed in a closed glasshouse chamber room where no other plants were present in the same room. The temperature and the ventilation of the chamber were regulated by fans, an automatic window-opening system, and manually operated heating elements. More precise control of environmental parameters were not implemented, to maintain conditions similar to a realistic working glasshouse. A measurement set-up was prepared on a raised bench in the glasshouse, with the data acquisition hardware (gas sensor boards and cameras) placed inside a plastic growing tent. Once the plants started producing fruits, individual strawberry plants were placed inside the tent for measurement. The three sensor boards were positioned on a raised platform inside the tent, approximately 10–15 cm from the edges of the strawberry pot. This positioning allowed the sensors to be in close proximity to the fruits, which overhang the sides of the pot (Figure 3a). The tent was used to shield the plants from the air movement generated by the fans inside the glasshouse chamber and to also separate it from the other strawberry plants. The plastic tent had a window open on one side, so this case-study was considered semi-open-air, but the tent offered some protection against the environmental factors. The final placement of the data acquisition platforms and measured plants is shown in Figure 2b. The data collection was run between March 7 and April 16 in 2025.

In this case-study, three sensor boards were placed on a small elevated platform to lie in close proximity to the strawberry fruits, and the acquisition boards were configured to run without supervision continuously. One board was configured to run the shorter acquisition program every 30 min (${\mathrm{DAQ}}_{10}$), while another board was running the longer acquisition program once every hour (${\mathrm{DAQ}}_{48}$). A third acquisition board was set-up to follow an acquisition protocol of constant 5 V heating applied (${\mathrm{DAQ}}_{cont}$). The two cameras were also set-up inside the tent, monitoring the plants from a front angle, taking an image every 30 min. Examples of the acquired data are illustrated in Figure 3.

Ripe and unripe strawberry plants were alternated every few days inside the tent. A total of 12 strawberry plants were grown, but only a subset of those plants produced fruits and were used for the data collection. Over the course of the data collection the plant inside the tent was swapped 11 times, leaving it inside the tent for a varying length of time (few days to a week) each time. Whenever the plant was exchanged, the sensor boards were adjusted on the raised platform to ensure that the sensors were close to the fruits. The plastic tent was lifted off the plant and the sensor board setup every time the strawberry plant was replaced, but the data collection setup was left undisturbed between. This continued for the duration of 1 month, until all the plants finished producing fruits.

The resulting odour dataset and its properties are summarised in Table 5, and the collected image dataset is summarised in Table 6. In total, 1167 odour samples were acquired with ${\mathrm{DAQ}}_{10}$. Out of these 1167, the 326 samples at the beginning were affected by contamination (residues left by condensation) of the sensor board, and because of that, those samples were filtered out. This resulted in 841 samples total, out of which 319 were ripe and 522 were unripe samples. With ${\mathrm{DAQ}}_{48}$ 661 samples were acquired, 374 ripe and 287 unripe samples. With ${\mathrm{DAQ}}_{cont}$ 26,366,271 data points were acquired in total. Furthermore, although a humidity and temperature sensor were present on all sensor boards, these sensors malfunctioned for ${\mathrm{DAQ}}_{48}$ and ${\mathrm{DAQ}}_{cont}$. Because temperature and humidity data was only available with ${\mathrm{DAQ}}_{10}$, it was not used in most subsequent processing of the data, but an analysis is shown in later sections for ${\mathrm{DAQ}}_{10}$.

## 3. Methods

### 3.1. Processing Odour Modality

Multiple machine learning models were selected for the odour data, following the most commonly used methods in odour recognition literature [49]. From the classical machine learning algorithms, random forest (RF), support vector machine (SVM), gradient boosted decision trees (XGB) and logistic regression (LR) were selected. These methods were chosen because of their ability to generalise well on small datasets, and they were trained on extracted features. Additionally, a multi-layer perceptron (MLP), a 1D convolutional neural network (1D-CNN), a simple stacked long-short term memory (LSTM) network and an extreme learning machine (ELM) were selected to also evaluate neural networks on the data. The 1D-CNN and LSTM were chosen for their ability to learn to extract features from the raw data, as well as for their ability to capture inter-sensor and temporal patterns of the response signal. Both the MLP and ELM were trained using extracted features. The MLP was selected for its ability to learn highly-non-linear patterns in the data, while the ELM was included due to its reported success in prior works [50,51,52]. Furthermore, for data visualisation, principal component analysis (PCA) was used as the dimensionality reduction algorithm.

**Figure 4:**
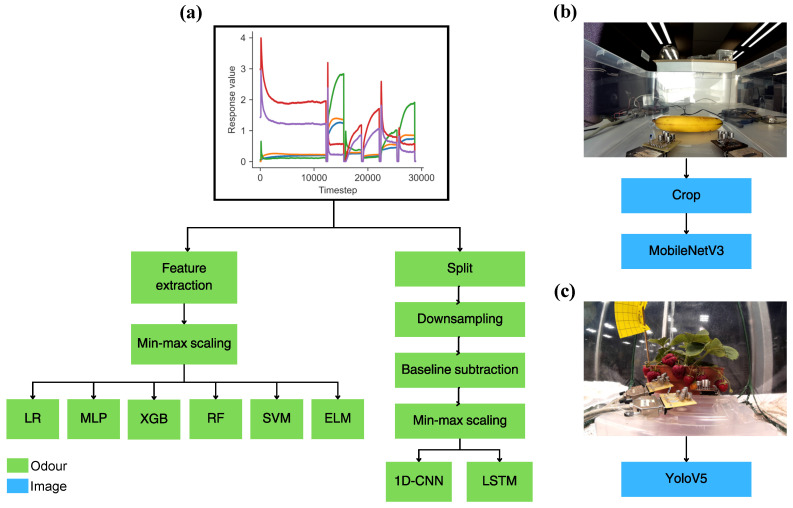
The pre-processing steps and ML methods applied on the data. (**a**) Odour modality (both banana and strawberry data). (**b**) Image modality (banana data). (**c**) Image modality (strawberry data).

The pre-processing and feature extraction steps and selected models are summarised in Figure 4. The raw response data $R\in {ℜ}^{S\times T}$ (where *T* is the signal length and S=5 is the number of sensors) first was split into the 5 modulation levels, resulting in the data $X_{7V}$,$X_{5.5V}$,$X_{5V}$,$X_{6V}$,$X_{6.5V}\in {ℜ}^{S\times M}$, where *M* is the length of each modulation level. For the classical machine learning models features were extracted from this data to reduce dimensionality. Following a similar feature extraction strategy to the one defined in [10], the maximum response value at 7 V modulation level (1), the difference value at 7 V modulation level (2), and the maximum of the exponential weighted moving average (EWMA) smoothed signal (3) at three alpha values ($\alpha_{1}=0.1$, $\alpha_{2}=0.01$, $\alpha_{3}=0.001$) were extracted for all sensors and all modulation levels. The features were min-max scaled (5) before being inputted to the models. This resulting feature vector f contained 2×5+3×5×5=85 values (4). In some cases a smaller feature set, containing only the maximum and difference values at the 7 V modulation stage (10 features), was also evaluated. Two of the neural networks, CNN and LSTM, used the whole response signal as input. For the CNN and LSTM, the raw response signal again was split by modulation levels. Each resulting signal was then downsampled to a length of 100 and 300 data points for ${\mathrm{DAQ}}_{10}$ and ${\mathrm{DAQ}}_{48}$ respectively, and baseline subtraction (6) as well as min-max scaling (7) was applied on the data.

(1) $$m_{7V}^{\left(s\right)}=\max_{1\leq m\leq M}\left(X_{7V}\left[s,m\right]\right)$$

(2) $$d_{7V}^{\left(s\right)}=\max_{1\leq m\leq M}\left(X_{7V}\left[s,m\right]\right)-\min_{1\leq m\leq M}\left(X_{7V}\left[s,m\right]\right)$$

(3) $$\begin{array}{c} {\hat{X}}_{v}^{\left(\alpha \right)}\left[s,m\right]=\frac{\sum_{i=1}^{m}{\left(1-\alpha \right)}^{m-i}X_{v}\left[s,i\right]}{\sum_{i=1}^{m}{\left(1-\alpha \right)}^{m-i}}\\ {\mathrm{ewma}}_{v}^{\left(s,\alpha \right)}=\max_{1\leq m\leq M}\left({\hat{X}}_{v}^{\left(\alpha \right)}\left[s,m\right]\right) \end{array}$$

(4) $$f=\left[m_{7V}^{\left(1\right)},m_{7V}^{\left(2\right)},\ldots ,m_{7V}^{\left(S\right)},d_{7V}^{\left(1\right)},\ldots d_{7V}^{\left(S\right)},ewma_{7V}^{\left(1,\alpha_{1}\right)},\ldots ewma_{6.5V}^{\left(S,\alpha_{3}\right)}\right]$$

(5) $${\hat{f}}_{i}=\frac{f_{i}-\min\left(f_{i}\right)}{\max\left(f_{i}\right)-\min\left(f_{i}\right)}$$

(6) $$\begin{array}{c} \;\mathrm{baseline}\left[s\right]=\frac{1}{5}\sum_{k=0}^{4}X\left[s,k\right]\\ \hat{X}\left[s,m\right]=X\left[s,m\right]-\mathrm{baseline}\left[s\right] \end{array}$$

(7) $${\hat{X}}_{v}\left[s,m\right]=\frac{X_{v}\left[s,m\right]-\min_{1\leq n\leq M}\left(X_{v}\left[s,n\right]\right)}{\max_{1\leq n\leq M}\left(X_{v}\left[s,n\right]\right)-\min_{1\leq n\leq M}\left(X_{v}\left[s,n\right]\right)}$$

Data from ${\mathrm{DAQ}}_{cont}$ was processed using a different approach, as the usual feature extraction techniques based on the response curve are not applicable on continuously acquired data. ${\mathrm{DAQ}}_{cont}$ data was split into 10 min non-overlapping segments, from which simple time-domain statistical features were extracted, a method that is commonly used in time-series data processing [53,54]. For each sensor, the mean, standard deviation, maximum, minimum, and root mean square values were computed within each window, resulting in a total of 25 features. As with the feature vectors for ${\mathrm{DAQ}}_{10}$ and ${\mathrm{DAQ}}_{48}$, all features were scaled using min-max normalization. For the CNN and LSTM models, the raw data was downsampled to a length of 100 data points and scaled using a min-max scaler.

**Figure 5:**
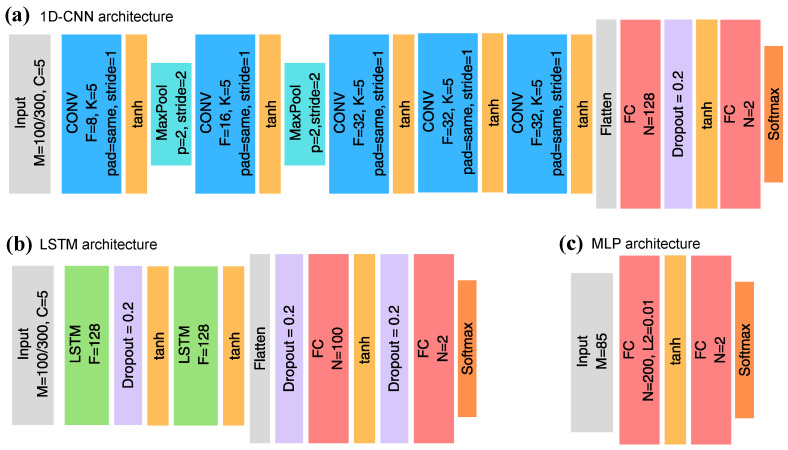
The neural network architectures used for processing the odour modality. (**a**) 1D-CNN architecture, based on [55]. (**b**) The stacked LSTM architecture. (**c**) The 3-layer MLP architecture.

The MLP network was used with one input, one hidden, and one output layer (Figure 5c). The 1D-CNN network architecture follows the one defined in [55], shown on Figure 5a). The LSTM was defined as a simple stacked LSTM architecture (Figure 5b). The neural networks (1D-CNN, LSTM, MLP) were trained with the cross-entropy loss-function and Adam optimizer, using early stopping during training. Batch size was set to be varying between 4 and 32. For all models, classical ML and NN, class weighting was enabled to counteract the imbalance in the dataset. For the SVM and the RF, hyperparameter-optimisation was used. Cross-validation training was used to evaluate the models on the training dataset (folds = 10). Uniquely, the strawberry odour data was also evaluated using leave-K-block-out cross-validation strategy [56], to counteract the strong temporal correlation present between the data and the labels. Random seeds were fixed for repeatability, and all training and testing was run 25 or 50 times with different random seeds, and the mean results are reported, to account for the random initialisations of the ML and NN models. For all implementations Python 3 was used, with scikit-learn (1.5.2) used for the classical ML models, TensorFlow (2.19.0) for the neural networks, and Python-ELM [57] for the ELM. The odour samples were labeled based on the image data, matched by their timestamps.

Where temperature and humidity values were used in the analysis, they were incorporated into the models with the following two methods. First, temperature and humidity values were used as additional input features to the MLP, LR, XGB, RF, SVM, and ELM models, where the values were concatenated to the feature vector f prior to scaling. The second approach corrects the raw response signal $V_{out}$ based on reference measurements at given humidity/temperature values provided in the sensor datasheets. The datasheets provide measurements for $R_{S}/R_{0}$ at selected humidity and temperature values, which is defined as the ratio between the measured response in resistance ($R_{S}$) and the sensor response under the reference condition 20 °C/65 RH% ($R_{0}$). A surface was fitted to these measurements using a radial basis function (RBF) to estimate a correction factor α for any humidity-temperature value pair. The corrected sensor response $V_{\tilde{out}}$ was acquired using (8), where the circuit voltage $V_{C}$ was 5 V. This correction was only applied to sensor 2–5, as this information was not available for sensor 1 in the datasheet.

(8) $$V_{\tilde{out}}=\frac{V_{C}}{\left(\frac{V_{C}}{V_{out}}-1\right)*\frac{1}{\alpha }+1}$$

### 3.2. Processing Image Modality

Two deep learning models were chosen for the banana and the strawberry images. The banana images were treated as a classification problem, and MobileNetV3 [58] was fine-tuned after initialising with ImageNet [59] weights, using the PyTorch (2.4.1) implementation [60]. For fine-tuning, 362 images in total from the banana ripening training data were used, and the images from test batch 1 were used as the validation dataset. The images were cropped and normalised, and random augmentations (resizing crop, flipping, contrast) were applied on the images.

The strawberry images were processed with an object detection algorithm, for which YoloV5 [61] was chosen, using the implementation by Ultralytics (7.0). The strawberry data was treated as object detection, as there are multiple strawberries growing on one plant at any given time and they often do not ripen at the same time. Then, the strawberry detection was reduced to a ripe/unripe classification, based on the following process. Bounding boxes with low confidence were filtered out, and overlapping bounding boxes were merged with non-maximum suppression. Then, images were labelled as “ripe” when any ripe strawberry was found on the image, or they were labelled as “unripe” when no strawberry or only unripe strawberries were found. The YoloV5 model was initialised with ImageNet weights and it was fine-tuned on a publicly available strawberry detection dataset [62]. To emphasise this, the YoloV5 was not trained nor fine-tuned on any of the data collected in this study. The collected images were hand-annotated with the bounding boxes for YoloV5, and was used for testing only.

It should also be noted that both MobileNetV3 and YoloV5 were chosen for their computational efficiency. As the target application would be deployed in-situ, compatible algorithms were selected for their suitability of running on embedded systems.

### 3.3. Decision Fusion of Modalities

**Figure 6:**
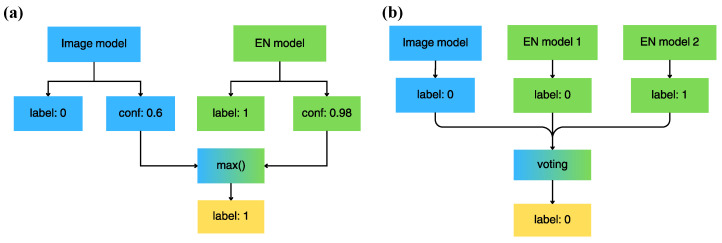
Two late fusion strategies were adopted. (**a**) Maximum confidence fusion. (**b**) Majority voting.

Two late fusion strategies were followed to combine the decisions of the odour and the image models: a maximum decision fusion and a majority voting strategy. The two strategies are illustrated on Figure 6. A maximum decision strategy combines the decision of two classifiers by selecting the label with the higher confidence (e.g., the higher softmax score). Consequently, only models for which decision confidence is available can be fused with this strategy. Majority voting assigns the majority label given by multiple classifiers. Therefore, majority voting can only be applied on a voting ensemble of more than two models.

### 3.4. Metrics

The models were evaluated using the following metrics. All models were compared by their accuracy (9), and precision (10) and recall (11) metrics were also used to observe the effect of the imbalanced nature of the datasets. Results on the strawberry ripening data was also measured by the F1 score (12). Furthermore, the performance of the object detection model (YoloV5) is also reported by the mean average precision (mAP) scores, namely the mAP50 and the mAP50-95 scores, which observes the mAP at IoU (13) 0.5 and 0.5–0.95.

(9) $$accuracy=\frac{\mathrm{number}\;\mathrm{of}\;\mathrm{correct}\;\mathrm{predictions}}{\mathrm{total}\;\mathrm{number}\;\mathrm{of}\;\mathrm{predictions}}$$

(10) $$precision_{c}=\frac{\mathrm{number}\;\mathrm{of}\;\mathrm{correct}\;\mathrm{predictions}\;\mathrm{for}\;\mathrm{class}\;c}{\mathrm{number}\;\mathrm{of}\;\mathrm{predictions}\;\mathrm{for}\;\mathrm{class}\;c}$$

(11) $$recall_{c}=\frac{\mathrm{number}\;\mathrm{of}\;\mathrm{correct}\;\mathrm{predictions}\;\mathrm{for}\;\mathrm{class}\;c}{\mathrm{number}\;\mathrm{of}\;\mathrm{instances}\;\mathrm{of}\;\mathrm{class}\;c}$$

(12) $$F1=2\times \frac{\mathrm{precision}\times \mathrm{recall}}{\mathrm{precision}+\mathrm{recall}}$$

(13) $$\mathrm{Intersection over Union}\left(\mathrm{IoU}\right)=\frac{\mathrm{Area}\;\mathrm{of}\;\mathrm{bounding}\;\mathrm{box}\;\mathrm{intersection}}{\mathrm{Area}\;\mathrm{of}\;\mathrm{bounding}\;\mathrm{box}\;\mathrm{union}}$$

## 4. Results on Odour Modality

### 4.1. Results on Case-Study 1: Banana Ripening

**Figure 7:**
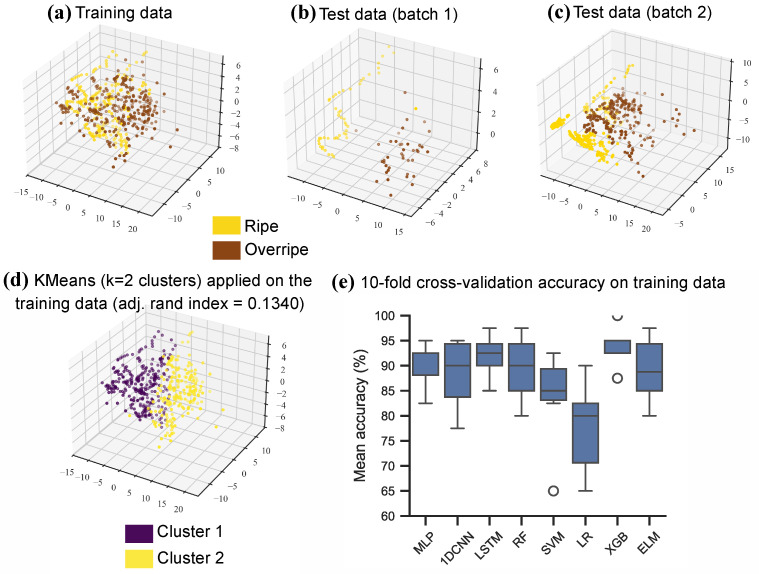
Visualisation of data from ${\mathrm{DAQ}}_{10}$ in the open-air banana case study and visualisation of results of the ML models. (**a**–**c**) PCA plots of the training and test datasets. (**d**) KMeans (k = 2 clusters) applied on the PCA transformed training dataset. (**e**) Box-plot of the 10-fold cross-validation accuracy results of the ML models on the training dataset.

The PCA plots for the banana data from ${\mathrm{DAQ}}_{10}$ (Figure 7a–c) showed poor separation of the classes on the training dataset, and showed stronger separation on test batch 1 and test batch 2. Similarly, the KMeans algorithm evaluated on the training dataset did not perform significantly better than random guessing, achieving an adjusted rand index of 0.1340 (Figure 7d). In contrast, most classical ML and NN models achieved good results on the training dataset when following a 10-fold cross-validation evaluation approach (Table 7 and Figure 7e). All of the models (except SVM) achieved close to 90% mean accuracy, where XGB achieved the highest mean cross-validation accuracy (93%), and the LSTM and the MLP achieved the second (91.50%) and third highest mean accuracies (90.50%).

**Figure 8:**
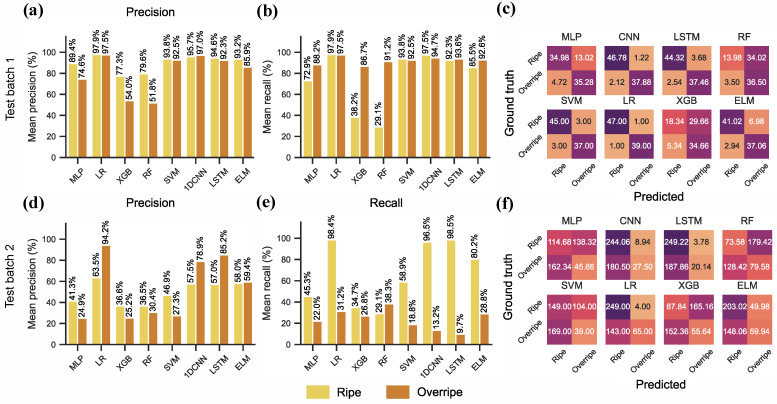
Visualisation of the class-wise ML results on the banana case-study data from ${\mathrm{DAQ}}_{10}$. All results are reported as the mean over 50 runs. (**a**–**c**) Class-wise precision and recall plots, and confusion matrices for test data batch 1. (**d**–**f**) Class-wise precision and recall plots, and confusion matrices for test data batch 2.

The results on the hold-out test datasets are shown in Table 8. Evaluation on test batch 1 demonstrated comparable to or better results than the cross-validation scores for the LR, SVM, CNN, ELM and LSTM models, while the MLP, XGB and RF models underperformed. On test batch 1, LR and CNN achieved the highest test accuracy with 97.72% and 96.20% respectively. The tree-based methods, RF and XGB, significantly underperformed with 57.36% and 60.22%. LR, CNN, LSTM, ELM and SVM achieved very good precision and recall (Figure 8a,b), above 90% for both classes. Models also were more likely to misclassify ripe samples as overripe samples, as shown on the confusion matrixes (Figure 8c). Accuracy results on test batch 2 were significantly lower compared to both test batch 1 and cross-validation results. Most models failed to achieve better than random guess (Table 8), with only LR achieving better than random chance with 68.11% accuracy. All models achieved poor recall (Figure 8d,e), and only the LR achieved adequate precision (ripe—63.5%, overripe—94.2%). All other models showed severely imbalanced results. Observing the confusion matrix confirms this (Figure 8f), most models (MLP, CNN, LSTM, SVM, LR, ELM) were more likely to misclassify overripe samples as ripe, while RF and XGB did the opposite.

**Figure 9:**
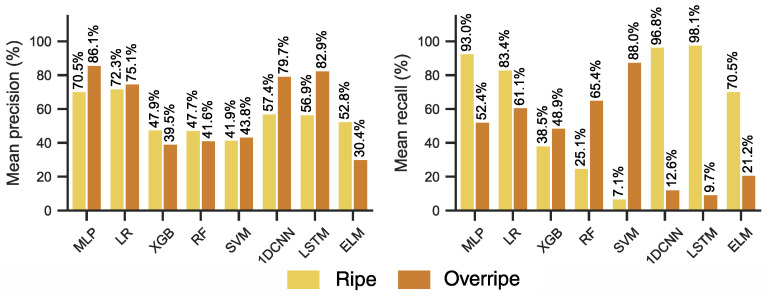
Precision and recall visualised for the open-air banana ripening test data (batch 2) when models were trained on the small feature-set. Results are reported as the mean over 50 runs.

The results of the models trained on extracted features were influenced by the feature selection method (Table 9). When testing on test batch 2, using the smaller feature set largely improved the accuracy of MLP (34.78% to 74.68%), and moderately improved the LR (68.11% to 73.31%), XGB (31.12% to 43.20%), RF (33.22% to 44.30%) and SVM (40.78% to 43.60%). Class-wise precision and recall was also higher when trained on the smaller feature set (Figure 9), although the models still showed bias towards assigning “ripe” label to samples. On test batch 1, the results showed the opposite trend, with models achieving higher accuracy when trained on the larger feature set.

Furthermore, using the 6.5 V modulation stage as the input to the CNN and LSTM was decided empirically, and the results of the 1D-CNN on test batch 1 of the banana ripening data over all modulation levels is shown in Table 10. Interestingly, the accuracy was mostly increasing with the modulation levels e.g., the first modulation level (7 V) yielded the worst accuracy with 60.79% and the last modulation level (6.5 V) yielded the best accuracy result with 96.20%.

Some of the results of the ML models can be explained by the experiment set-up. The labels of the ripe and overripe banana were determined based on their visual appearance. As ripening is a gradual process, there were samples that could have been classified as either ripe or overripe. Results confirm this, where most misclassification happened by classifying ripe samples as overripe. Furthermore, the gap between test results on test batch 1 and 2 can be likely explained by either the different positioning of the sensors, or the sensor drift introduced to the data by the time gap in acquisition. Given the open-air setting of the data acquisition, the sensor drift could be also second-order [10] in nature, having environmental origins. On the other hand, the poor separation observed in the training dataset with PCA and KMeans can likely be attributed primarily to the variable positioning of the sensors during its collection, as test batch 2 showed significantly better separation (the good separation in test batch 1 likely reflects its small sample size). Due to this poor separation with PCA, it was expected that KMeans would not be able to cluster it well.

However, unexpected results were the accuracy of the models on test batch 2 with the different feature selection. Models performed better when trained on the small feature set, which feature set only had the maximum and difference response values. This could mean that the amplitude of the response is more robust against distribution shift than transient features, or that the smaller size of the model trained on the small feature set acted as regularisation to prevent over-fitting on the training data. The results of evaluating the temperature modulation levels on the 1D-CNN was also unexpected. It was expected that the 7 V modulation stage, which happens right after the pre-heating period, would be more stable. However, the 7 V temperature modulation level resulted in the worst accuracy, while the last modulation level (6.5 V) achieved the best accuracy.

### 4.2. Results on Case-Study 2: Strawberry Ripening

#### 4.2.1. Results for ${\mathrm{DAQ}}_{10}$ and ${\mathrm{DAQ}}_{48}$

**Figure 10:**
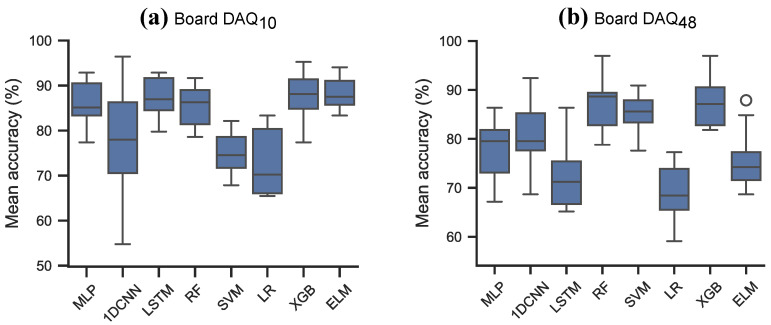
10-fold cross-validation scores on the strawberry odour datasets. (**a**) Data from ${\mathrm{DAQ}}_{10}$. (**b**) Data from ${\mathrm{DAQ}}_{48}$.

As the strawberry dataset was not pre-split into training and hold-out test sets, the models were evaluated with two cross-validation strategies: a stratified 10-fold cross-validation strategy, and a leave-5-day-out cross-validation strategy (similar to the leave-one-block-out strategy described in [56]). The results of the 10-fold cross-validation strategy is shown in Table 11 and Figure 10. The models trained on data from ${\mathrm{DAQ}}_{10}$ on average achieved higher cross-validation results compared to when models were trained on data from ${\mathrm{DAQ}}_{48}$, measured by the mean accuracy. The two tree-based methods, XGB and RF achieved very good accuracy on both datasets, appr. 88% and 85% respectively. Generally, comparable results were achieved by the classical machine learning models (3.54% mean difference) and larger differences for the neural network models (10.35% mean difference) between the two data source. However, multiple models trained on data from ${\mathrm{DAQ}}_{10}$ had large spreads (Figure 10a), while models trained on ${\mathrm{DAQ}}_{48}$ (Figure 10b) achieved smaller spread.

**Figure 11:**
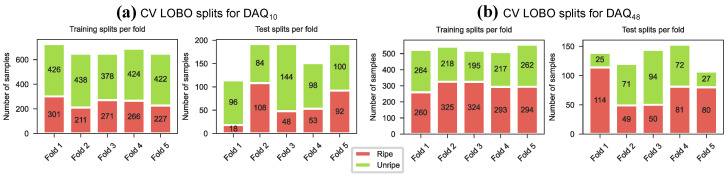
Split of training and test data in the LOBO cross-validation. (**a**) Data from ${\mathrm{DAQ}}_{10}$. (**b**) Data from ${\mathrm{DAQ}}_{48}$.

**Figure 12:**
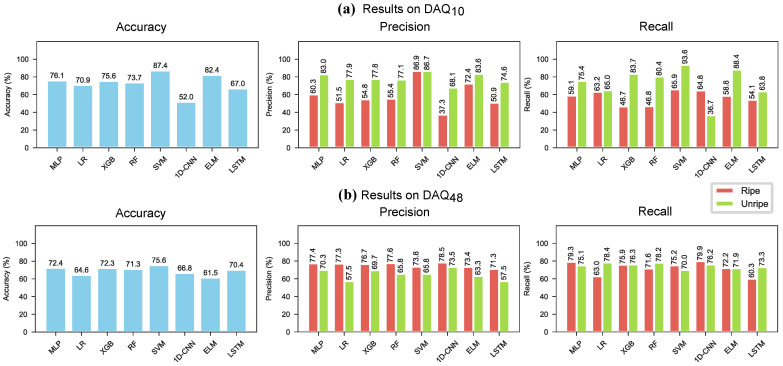
Results on the strawberry data following a leave-5-day-out cross-validation strategy, averaged over 10 runs for each fold. (**a**) Results on ${\mathrm{DAQ}}_{10}$. (**b**) Results on ${\mathrm{DAQ}}_{48}$.

**Figure 13:**
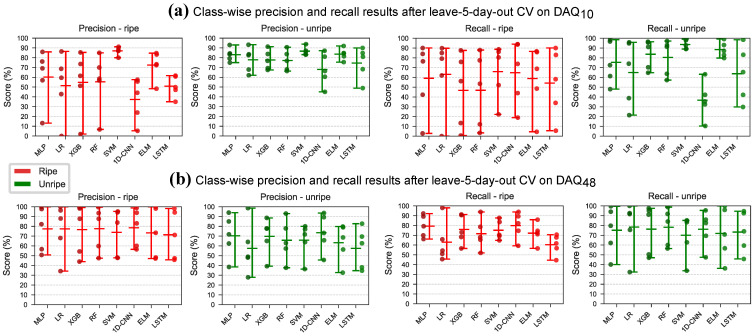
The spread of the class-wise precision and recall when evaluating on the strawberry dataset with the leave-5-day-out cross-validation strategy. (**a**) Results on ${\mathrm{DAQ}}_{10}$. (**b**) Results on ${\mathrm{DAQ}}_{48}$.

The models were also evaluated with a leave-5-day-out strategy, similar to the leave-one-block-out (LOBO) cross-validation. On each LOBO split the models were trained 10 times to account for random initialisation. The LOBO splits are illustrated in Figure 11 and the mean accuracy, precision and recall results are shown in Table 12 and Figure 12. The leave-5-day-out cross-validation resulted in lower accuracy than the regular cross-validation strategy for both data sources. Generally, models trained on data from ${\mathrm{DAQ}}_{10}$ achieved higher accuracy than models trained on data from ${\mathrm{DAQ}}_{48}$. SVM achieved the highest accuracy on both datasets, with 87.4% on ${\mathrm{DAQ}}_{10}$ and 75.6% on ${\mathrm{DAQ}}_{48}$. The 1D-CNN model achieved the highest F1 score on ${\mathrm{DAQ}}_{48}$ with 75.6%. Illustrating the spread of the class-wise precision and recall over the 5 folds 400 and 10 repeats (Figure 13) on the other hand showed that models trained on ${\mathrm{DAQ}}_{10}$ favoured data belonging to the unripe class, while models trained on ${\mathrm{DAQ}}_{48}$ were mostly balanced for all models. Furthermore, precision and recall results on ${\mathrm{DAQ}}_{10}$ had very large spread, especially for the ripe class (Figure 13a), while ${\mathrm{DAQ}}_{48}$ again showed more balanced learning (Figure 13b).

#### 4.2.2. Effect of Humidity and Temperature on ${\mathrm{DAQ}}_{10}$

Temperature and humidity values were available for ${\mathrm{DAQ}}_{10}$, therefore an ablation study was performed to see the effect of temperature and humidity on the data. The results are shown in Table 13. Using the temperature and humidity values as features resulted in slightly increased mean cross-validation accuracy for MLP (+1.77%), LR (+3.8%), XGB (+1.54%), and RF (1.06%). XGB achieved the highest mean accuracy (89.18%) out of all experiments on ${\mathrm{DAQ}}_{10}$, when using the temperature and humidity values as features. Correcting the raw sensor responses did not result in any significant difference for any of the models, except for the ELM, which achieved worse results with correction (−11.77% decrease).

#### 4.2.3. Results for ${\mathrm{DAQ}}_{cont}$

**Figure 14:**
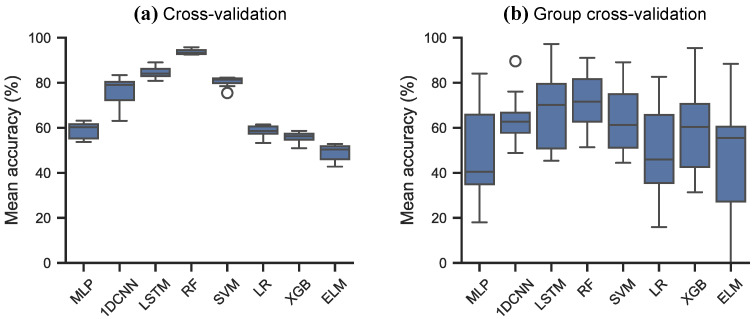
Cross-validation scores on the ${\mathrm{DAQ}}_{cont}$ strawberry odour dataset. (**a**) Stratified 10-fold cross-validation. (**b**) Stratified 10-fold group cross-validation.

The cross-validation results of the models on ${\mathrm{DAQ}}_{cont}$ are shown in Figure 14 and in Table 14. Similarly to the other datasets, two cross-validation strategies were applied; a stratified 10-fold cross-validation with shuffling, and a stratified 10-fold group cross-validation, where the data was grouped into 24-hour long groups. The RF (93.67%), LSTM (84.65%), SVM (80.52%) and CNN (76.62%) achieved high cross-validation accuracy, while all the other models achieved poor (<60%) mean accuracy. When following the stratified 10-fold group cross-validation approach, most models underperformed, with only RF achieving moderately good results (72.10%). Furthermore, all models had very high variance, showing that models struggle to generalise on this dataset.

#### 4.2.4. Summary of the Strawberry Results

These results show the importance of following a LOBO approach when evaluating data with many repeat measurements. Even though models trained on data from ${\mathrm{DAQ}}_{10}$ achieved higher accuracy when following LOBO, and much higher accuracy with cross-validation, models trained on ${\mathrm{DAQ}}_{48}$ are more balanced and consistent across all models and all folds. One reason for this is the imbalanced nature of the data. After data cleaning, data from ${\mathrm{DAQ}}_{10}$ was heavily imbalanced and it was much less diverse. As opposed to that, data collection from ${\mathrm{DAQ}}_{48}$ was more successful, such that more measurements were taken for a longer period of time. The length of acquisition also may explain this difference in performance. A shorter acquisition length may be more influenced by short-term environmental factors, whereas the sensors have more time to become saturated when following a longer acquisition with the longer pre-heating. Compared to both, models significantly underperformed on ${\mathrm{DAQ}}_{cont}$, which indicates that inducing the transitorial response with temperature modulation is vital to obtain robust features in such an open-air setting.

Evaluation of the ML models on the strawberry dataset showed that there is potential in using the sensors in a temperature-modulated way in an open-air setting to recognise the smell of ripe strawberries. However, environmental changes during this data collection introduced substantial short-term noise in the measurements. When a simple cross-validation strategy was applied, temporally close measurements were present in both training and test datasets, producing overly optimistic results. Therefore, a leave-k-day-out evaluation was essential to mitigate this effect. In future works, a separate hold-out test dataset would enable a more robust evaluation, although this was not possible here due to the limitations of the data collections.

## 5. Results on the Image Modality

### 5.1. Results on Case-Study 1: Banana Ripening

**Figure 15:**
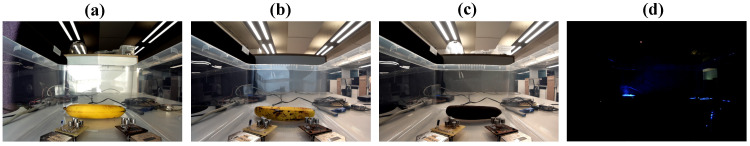
Illustrative images of the banana images, taken from the training dataset. (**a**,**b**) Example image of a ripe banana. (**c**) Example image of an overripe banana. (**d**) Image taken at night.

The banana ripening case-study was conducted in an indoors office environment, where during the daytime both artificial overhead light and natural light through the windows lit the setup. This provided good lighting conditions between 10–18 h each day. Because of this, the auto-exposure of the cameras worked well, and resulted in clear well-lit images during the day. Trivially, at night no usable images were obtained, as the lights were turned off. Sample images are shown in Figure 15.

The results of MobileNetV3 on images from test batch 2 are summarised in Table 15. The results in the table are shown for images that were taken in well-lit conditions, and results are also shown projected to all data, including data that was acquired at night. In that case, night images were treated as missing as the image model cannot be expected to do reliable classification. In the accuracy calculation, the night images were counted as misclassification. The results show that MobileNetV3 can classify the images with almost 100% accuracy on the daylight images. This was expected, as image models generally perform very well on images similar to the training (and/or fine-tuning) data. On the other hand, when the results were projected to all data, including night images, MobileNetV3 achieved only 46.20% accuracy.

### 5.2. Results on Case-Study 2: Strawberry Ripening

**Figure 16:**
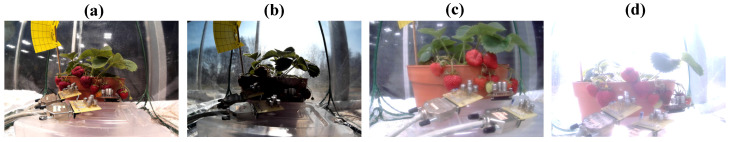
Illustrative images from the strawberry image data. (**a**) Well-lit image of ripe strawberries, taken with Camera 1. (**b**) Strongly backlit images of ripe strawberries, taken with Camera 1. (**c**) Well-lit image of ripe strawberries, taken with Camera 2. (**d**) Strongly backlit images of ripe strawberries, taken with Camera 2.

Images collected in the strawberry ripening case-study were affected by the harsh lighting changes inside the glasshouse throughout the day. Some example images are shown in Figure 16. Strong backlight was found to be the main challenge, as it is difficult to compensate algorithmically or to mitigate physically in a glasshouse. It affected a large portion of the images, as most daylight images that were taken in sunlight. Camera 1 produced images with underexposed foreground (Figure 16b), while camera 2 produced overall overexposed images in strong backlight. Still, camera 1 produced better quality and overall well-exposed images in other circumstances, therefore only images from camera 1 were evaluated with the object detection model.

The YoloV5 was first fine-tuned on a publicly available strawberry dataset, and the model was evaluated on the images collected during the case-study from camera 1. The performance metrics across the 1717 images are reported in Table 16 and Table 17, with and without confidence thresholding respectively. The model demonstrated marginally higher recall for unripe strawberries (43.80%) relative to ripe instances (38.80%), and a substantially higher precision (73.60% vs. 63.90%). These results show that the model was better at discriminating and assigning higher confidence scores to unripe fruits. However, with a higher confidence thresholding, this difference between ripe and unripe scores became negligible, resulting in 97.00% and 94.70% precision and 14.00% and 12.10% recall for unripe and ripe respectively. Because the primary objective was to identify reliably ripe strawberries, precision was prioritized in order to minimize false positives, and recall was secondary.

**Figure 17:**
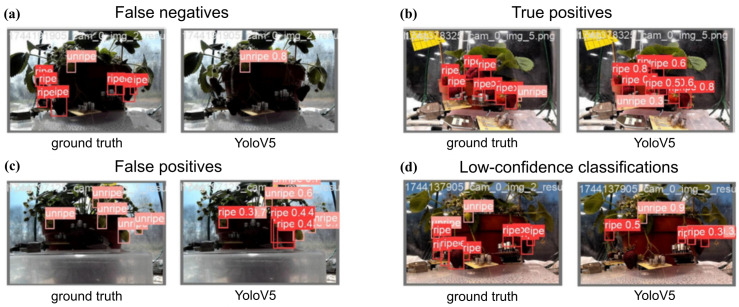
Examples of annotated ground-truth and YoloV5 prediction pairs on images from camera 1. (**a**) On images with strong backlight, Yolov5 has poor recall, resulting in many false negatives. (**b**) On well-lit images, YoloV5 performed well. (**c**) Under strong backlight, YoloV5 often produced false positives, confusing dark foreground for ripe strawberries. (**d**) Although under artificial light YoloV5 performed better than on daylight images, many bounding boxes were of low-confidence.

Looking at the object detection bounding boxes visualised on the images explains some of these results. Selected examples are shown in Figure 17. As it was expected, the models showed difficulties classifying images with strong backlight, particularly when classifying ripe fruits. Unripe fruits were more distinguishable even in strong backlight, therefore, object detection achieved better recall on them. Under favourable lighting conditions, the model detected ripe fruits more reliably. But even then, the confidence scores were lower for the ripe class, thus, the confidence thresholding removed a considerable number of true positives as well as false positives.

The object detection was mapped to a classification problem, by assigning a binary label at the image level based on whether ripe fruit is present on the image or not. An image was labelled as ripe if any ripe fruit was detected, and it was classified as unripe otherwise. Classification performance under this approach is summarised in Table 18. Across the 1717 images, YoloV5 detected objects on 533 of the images (31.04%). The accuracy measured on all images was 26.55%, while accuracy restricted to images with at least one detection was 85.55%. These results are aligned with the results in the previous section, that is the model had low recall but relatively high precision. To further investigate the effect of lighting conditions on the images, a subset of 498 images captured under artificial light was analysed, where all images taken in daylight and all images taken in the dark were filtered out. In this subset, strawberries were detected on 361 images (72.49%), out of which 344 were correctly classified (95.29%). These findings are in line with previous strawberry detection works [22,23], where others emphasised the role of consistent artificial lighting in achieving good accuracy.

## 6. Results of the Decision Fusion

### 6.1. Results on Case-Study 1: Banana Ripening

**Figure 18:**
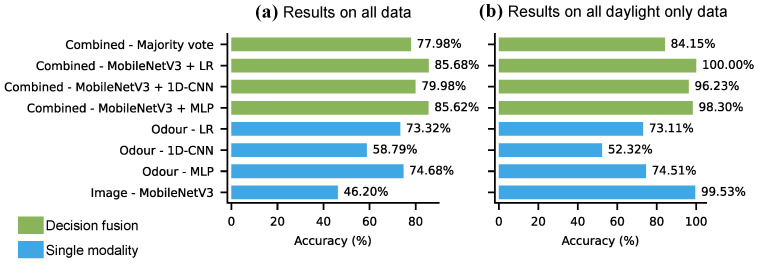
Combined results of MobileNetV3 and odour models on the banana ripening data (test batch 2). Results are shown both for daylight only and all data, averaged over 25 runs.

The decision fusion was evaluated only on test batch 2, as in the case of test batch 1 the fusion methods could not offer any improvement over the unimodal results. The results of maximum confidence fusion of MobileNetV3 with LR, MLP, and 1D-CNN models, and the majority voting (MobileNetV3, LR and MLP) is shown in Figure 18. These odour models were selected for fusion because they produced mostly consistent results in the analysis. The models were evaluated on all data (Figure 18a) and on daylight images only (Figure 18b).

When evaluating only on samples collected during the day (Figure 18b), the multimodal approach did not improve significantly on the accuracy of its unimodal components. The fusion of MobileNetV3 and LR achieved a perfect 100% accuracy, however the margin of improvement was less than 1% over the accuracy of MobileNetV3 (99.53%). Fusing MobileNetV3 with the MLP and 1D-CNN resulted in slightly worse accuracy (98.30% and 96.23% respectively) than using MobileNetV3 only (99.53%). Majority voting similarly resulted in worse accuracy (84.15%) than its unimodal components.

When evaluating on all data, the decision fusion methods all resulted in improvement over their unimodal components (Figure 18a). MobileNetV3 was not inferenced on images taken at night MobileNetV3, instead those samples were counted as misclassification, resulting in 46.20% accuracy. The odour models, LR, 1D-CNN and MLP, achieved 73.32%, 58.79% and 74.68% accuracy respectively. Following the maximum confidence fusion strategy LR and MobileNetV3 achieved the highest accuracy with 85.68% (12.36% increase). MLP and MobileNetV3 achieved almost the same accuracy with 85.62% (10.94% increase). Although the fusion of MobileNetV3 and 1D-CNN resulted in worse accuracy than the fusion of MobileNetV3 and other odour models (79.98%), the level of improvement was the highest here with 21.19% accuracy increase. Majority voting resulted in modest improvement (77.68% accuracy or 3.0% increase). This improvement can be attributed to the complementary information provided by the two modalities, where the models utilized the high-accuracy predictions of the MobileNetV3 when the image modality was available, and relied on the odour modality otherwise.

### 6.2. Results on Case-Study 2: Strawberry Ripening

**Figure 19:**
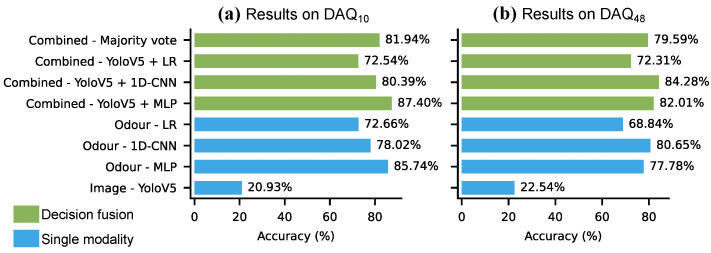
Combined results of YoloV5 and odour models on the strawberry dataset. Results are shown on data from both acquisition boards on all data (both daylight and night), averaged over the 10-fold of the cross-validation results.

Figure 19 presents the maximum confidence decision fusion results on the strawberry odour data. YoloV5 was combined with LR, 1D-CNN and MLP, and a majority voting of YoloV5, LR and MLP was also evaluated. YoloV5 was fused with the 10-fold-cross validation results of the odour models, and the decision fusion was evaluated on all samples (collected at both day and night) from the two odour data sources ${\mathrm{DAQ}}_{10}$ and ${\mathrm{DAQ}}_{48}$. The highest accuracy was achieved by YoloV5 + MLP on ${\mathrm{DAQ}}_{10}$ with 87.40% accuracy and by Yolov5 + CNN on ${\mathrm{DAQ}}_{48}$ with 84.28%, translating to +1.66% and +3.63% increase. The decision fusion approach consistently improved on the unimodal results, however, the accuracy gain was small. The reason for the small accuracy increase was that the YoloV5 performed poorly on the images (20.93% and 22.54%), therefore the improvement it could offer was limited.

Although decision fusion did not yield significant improvement on the strawberry data, the fusion of these two modalities presented another way to improve on the system. When examining the samples where both modalities agree (Table 19), it demonstrates that the decision fusion of modalities can be utilised to acquire labels that were likely to be correctly classified. For ${\mathrm{DAQ}}_{10}$ the 1D-CNN combined with YoloV5 achieved the highest agreement accuracy of 99.30%, and for ${\mathrm{DAQ}}_{48}$ the MLP and the YoloV5 with agreement accuracy of 99.12%. The two models agreed on 143 examples out of 841 from ${\mathrm{DAQ}}_{10}$, and 114 out of 661 from ${\mathrm{DAQ}}_{48}$. Both LR and MLP achieved comparable results on the data.

## 7. Discussion

It was demonstrated in the banana ripening case-study, that very high cross-validation accuracy and test accuracy can be achieved for in-distribution samples. This aligns with [28], where similarly good results were reported on the classification of banana ripening in a closed-sampling setting. This shows that in well-controlled environments, where there is a strong separation between the target odours, using the odour modality alone is enough to achieve very high accuracy. Additionally there are certain scenarios, where odour data was shown to be more advantageous than images. Such a scenario is when data is acquired in low-light or under dynamic lighting conditions. Odour data is unaffected by such conditions, whereas image models are sensitive to variable lighting, as also noted in the in-field strawberry detection literature [22,23].

However, acquiring odour data requires specialised equipment (EN, spectrometry) and expertise, unlike image acquisition. In controlled environments with minimal distribution shift and stable lighting conditions, image models alone can achieve very high accuracy; MobileNetV3 achieved 99.53% accuracy in the banana case-study when evaluated on daylight images. This raises questions about the benefit of incorporating a second modality alongside image data in scenarios similar to the banana ripening case-study. The results suggest that focus should be directed towards obtaining a large and diverse image dataset for fine-tuning.

The benefit of using a multimodal approach becomes clear when one looks at situations where both of the modalities have an innate shortcoming. This is typically the case when the system must make predictions at arbitrary times, under all environmental conditions. Image models degrade under low or variable lighting conditions, and MobileNetV3 achieved 46.20% accuracy when the test batch 2 results were projected across both day and night images. In the same case-study, odour models achieved only moderate performance due to the distribution shift of the dataset. However, the decision fusion strategy resulted in significant improvement (>10% for all fusion strategies) due to the complementary nature of the two modalities.

Furthermore, when observing cases where both modalities agree in the strawberry ripening case-study, another potential use of a multimodal approach emerges. In such cases we found that the labels tend to be correct, with as much as 99.30% accuracy. Although this constituted a small portion of the samples, this could be potentially used for automatic label generation (pseudolabeling) in domain adaptation or continual learning scenarios.

### 7.1. Considerations for In-Situ Deployment

In in-situ deployment, system response time is a vital consideration. For an electronic nose and camera system, it is mainly determined by the gas sensor acquisition cycle length, which takes several minutes (10–48 min in this work). On the other hand, imaging itself takes a few milliseconds, and image model inference on an embedded device typically takes a few seconds. Since fruit ripening happens gradually over several days, the time required for data acquisition and inference is negligible. With a 10 min acquisition cycle, the system could do hundreds of samplings per day.

Furthermore, system cost is a key factor in the commercial viability of deployment. The proposed electronic nose and RGB imaging system can be made cost-effective. RGB cameras are available across a wide price-range. The machine vision camera used in this work costs 179 USD, but lower-cost alternatives (e.g., Arducam or the Raspberry cameras) are available for under 100 USD. Figaro gas sensors typically cost less than 50 USD per sensor. Although in this work a more expensive MyRio board (appr. 1000 USD) with a Raspberry Pi 4b (appr. 50 USD) was used, lower-cost alternatives could be utilised instead, reducing the total cost of the system to a few hundred USD. However, integration onto a mobile platform would increase the cost significantly.

The algorithmic side of the system could be improved with better ML model selection methods. In this work, no single ML model emerged as consistently better than the rest. This opens up an avenue for investigating AutoML [63] techniques for this task, such as neural architecture search and Bayesian hyperparameter optimisation. However, such an approach would likely increase the complexity and cost of the system and would require cloud-computing infrastructure.

Based on these considerations, future works should focus on the following to improve real-life deployment feasibility. Lower-cost cameras may work adequately with better lighting and with improved exposure control. Additionally, the odour and image data acquisition could be integrated into a single, cost-effective platform. Further sensor selection and evaluation would also ensure that only sensors essential for the fruit ripening monitoring are used. Finally, shifting the monitoring setup from a one-fruit-at-a-time to a glass-house level setup could reduce the costs significantly.

### 7.2. Limitations of the Study

The banana ripening case-study had the following limitations. Between the two test batches the positioning of the sensors were changed as well as the data acquisition was separated by one month. Consequently, any difference observed in the distributions of the two batches could be attributed to either the placement of the sensors or to the time taken off from data acquisition. Also, the bananas were not ripening uniformly, therefore the length of measurement for each fruit was varying, introducing imbalance to the data.

Similarly in the strawberry case-study the dataset is imbalanced, due to the strawberry plants not ripening uniformly. Removal of the acquisition equipment from the glasshouse due to maintenance work and sensor board cleaning exacerbated this imbalance. Malfunction of the devices and contamination of the data resulted in further data loss. Furthermore, no separate hold-out test data was collected in the strawberry case-study. Because of this, the data was evaluated only by cross-validation strategies, whereas testing on a separate hold-out test dataset (like with the banana case-study) would give more reliable estimation of deployed performance.

The data from the strawberry case-study was also affected by the activities inside the glasshouse. Variations in humidity, watering of the plants, treatments applied, and human activity may all affect the quality of the collected data by introducing noise and variability that can not be attributed to the observed targets. The data collection was further limited by the relatively short-term time-frame for all case-studies, with a maximum of 1.5 months of data collection. Similar future studies should aim to collect data over multiple growing seasons, to observe the effect of these short-term disturbances, as well as the long-term sensor drift and environmental changes.

Lastly, we must note that the methodology used for data processing was not exhaustive. We are aware of the large corpus of literature in odour recognition models, as well as data fusion methodologies. Future works should aim for benchmarking more odour recognition models, as well as mid and early fusion strategies. Similarly, for object detection there are many alternatives in the literature. This work demonstrated that Yolov5 underperformed on images taken in challenging lighting. Other, larger object detection or image segmentation models may perform better on this dataset.

## 8. Conclusions

The introduction cited the possible gains that can be achieved by using a multimodal approach. We showed in this paper that odour-image multimodality results in three types of gains in such fruit ripening scenarios. In both open-air settings accuracy gain was achieved by a simple data fusion approach, increasing the accuracy by as much as 11%. Additionally, where no significant accuracy gain was achieved, an increase in confidence was observed. This was demonstrated by analysing the accuracy of the labels where both odour and image modality agreed, and it was shown to be as much as 99.30%. For both case-studies, a gain in completeness was demonstrated, showing that neither modality is consistently available at all times; therefore, using two modalities introduces greater reliability to the system. We also argued that this careful evaluation of possible gains from a multimodal approach is vital. Therefore, this work aims to encourage readers to explore the fusion of odour and image data in other application scenarios using metrics beyond classification accuracy.

Future works should aim at collecting data in a fully open set-up, following the semi-open-air set-up we followed in the strawberry ripening case-study. Furthermore, it is advisable that in such scenarios the entirety of the glass-house is to be monitored, instead of monitoring only a single plant. Also, here simple ripe/unripe and ripe/overripe binary classifications were investigated. However, in order to translate the research into commercial uses, more complex classifications should be investigated. Namely, a system that predicts the optimal ripening time of strawberries (or other fruits) could help with achieving more precise timing of the harvest, thus reducing the cost of growing fruits. Therefore, translating the scenario from classification to regression (time until optimal ripeness) is a research avenue that could be highly beneficial to precision agriculture.

## Figures and Tables

**Table 1:** Related works in odour-image multimodal fusion.

Paper	Application	Sensor	Image	Fusion	Method	EN/Image/Fusion Accuracy (%)
[35]	Assessing mutton freshness	10 MOX sensors (PEN3), sampling chamber type	Hyperspectral	Feature fusion	Input Modified CNN (IMCNN)	n/a (regression)
[36]	Beef freshness assessment	8 QCM sensors with polymer films (KSV 5000)	Multispectral images (VideometerLab)	Decision fusion	PLSR	-/-/100.00
[37]	Strawberry fungi infection identification	10 MOX sensors (PEN3), sampling chamber type	Lab-scale visible/near-infrared HSI system	Feature fusion	SVM	n/a (regression)
[38]	Green vegetable identification in food	10 MOX sensors (PEN3), sampling chamber type	Hyperspectral (camera and spectrometer) + lighting system	Data fusion	LDA	86.37/93.28/97.50
[39]	Moisture content prediction in thawed pork	6 MOX type sensor, sampling chamber type	Hyperspectral imaging with camera, spectrometer	Decision fusion	PLSR	n/a (regression)
[40]	Black tea fermentation monitoring	10 MOX sensors (PEN3), sampling chamber type	Camera + light source	Feature fusion	SVM	86.67/82.22/95.56
[41]	Tea quality identification	10 MOX sensors (PEN3), sampling chamber type	CMOS camera	Decision fusion	SVM	88.89/88.89/100.00
[42]	Detecting freshness of spinaches	7 MOX sensors, sampling chamber type	Camera + light source	Feature fusion	MLP	81.25/85.42/93.75
[43]	Tomato quality identification	10 MOX sensors, sampling chamber type	CCD camera + light source	Feature fusion	SVC	75.36/85.51/94.20
[28]	Banana ripeness classification	7 MOX type sensors and sampling chamber	CMOS camera	Feature fusion	PCA + kNN	98.10/99.05/100.00
[44]	Meat quality classification	CCS811 gas sensor in sampling chamber	CMOS camera	Feature fusion	CNN and DeepLab V3+ fused with 1D-CNN	-/-/99.44
[45]	Perfume and smoke identification	7 MOX type sensors in open-air	Thermal camera	Feature fusion	LSTM (odour) and CNN (image)	82.00/93.00/96.00

**Table 2:** The Figaro sensors used in the data acquisition and their sensitivity.

Numbering	Sensor	Sensitivity
Sensor #1	TGS2612	Methane, propane, iso-butane
Sensor #2	TGS2611	Methane, Natural Gas
Sensor #3	TGS2602	Air contaminants (VOCs, ammonia, H2S, etc.)
Sensor #4	TGS2600	Air contaminants (hydrogen, ethanol, etc.)
Sensor #5	TGS2620	Organic vapors (alcohol, solvent vapors)

**Table 3:** The temperature modulation patterns of $V_{H}$ applied on the heater element of the sensors, showing the sequence of voltages over time.

	$\overset{\;\mathrm{Time}\;(t)\;}{\to }$										
Length	5 V	0 V	7 V	0 V	5.5 V	0 V	5 V	0 V	6 V	0 V	6.5 V
${\mathrm{DAQ}}_{10}$	5 m	-	30 s	30 s	30 s	30 s	30 s	30 s	30 s	30 s	30 s
${\mathrm{DAQ}}_{48}$	20 m	30 s	300 s	30 s	300 s	30 s	300 s	30 s	300 s	30 s	300 s

**Table 4:** Summary of the open-air dataset of ripening bananas, collected with ${\mathrm{DAQ}}_{10}$.

Data	Ripe	Overripe	All
Training data	164	236	400
Test data batch #1	40	48	88
Test data batch #2	253	208	461

**Table 5:** Distribution of odour samples collected in the strawberry case-study.

Source	Ripe	Unripe	Total
${\mathrm{DAQ}}_{10}$	319	522	841
${\mathrm{DAQ}}_{48}$	374	287	661

**Table 6:** Summary of the RGB images collected in the strawberry case-study.

Source	Data Shape	Number of Images
Camera 1-See3CAM	[2304, 1536, 3]	1632
Camera 2-HD webcamera	[1920, 1080, 3]	1862

**Table 7:** Mean cross-validation results on the banana ripening training data from ${\mathrm{DAQ}}_{10}$ (%).

Data	MLP	LR	XGB	RF	SVM	CNN	ELM	LSTM
${\mathrm{DAQ}}_{10}$	90.50	77.25	**93.00**	89.25	84.50	88.74	89.25	91.50

**Table 8:** Mean test accuracy results (averaged over 50 runs) on the open-air banana dataset (%).

Data	Test Batch	MLP	LR	XGB	RF	SVM	CNN	ELM	LSTM
${\mathrm{DAQ}}_{10}$	1	79.84	**97.72**	60.22	57.36	93.18	96.20	88.72	92.93
${\mathrm{DAQ}}_{10}$	2	34.78	**68.11**	31.12	33.22	40.78	58.90	57.04	58.42

**Table 9:** Effect of feature selection on the accuracy in the banana case-study (%), averaged over 50 runs.

Features	Test Batch	MLP	LR	XGB	RF	SVM	ELM
Small	1	68.27	92.04	57.97	57.22	38.04	54.56
Large	1	79.84	**97.72**	60.22	57.36	93.18	88.72
Small	2	**74.68**	73.31	43.20	44.30	43.60	48.26
Large	2	34.78	68.11	31.12	33.22	40.78	57.04

**Table 10:** Results of the 1D-CNN algorithm based on the $V_{H}$ level applied, tested on open-air banana ripening data (test batch 1). Results are averaged over 20 runs. (%).

$V_{H}$	Accuracy (%)	Standard Deviation	Precision (%)		Recall (%)	
			Ripe	Overripe	Ripe	Overripe
7 V	60.79	0.0783	64.22	59.55	63.43	57.62
5.5 V	92.38	0.0019	94.65	90.65	91.25	93.75
5 V	94.94	0.0146	94.60	95.51	96.25	93.37
6 V	94.26	**0.0013**	**96.71**	92.23	92.70	**96.12**
6.5 V	**96.20**	0.0254	95.72	**97.02**	**97.45**	94.70

**Table 11:** Mean 10-fold cross-validation accuracy results on data from the strawberry case-study (%).

	MLP	LR	XGB	RF	SVM	CNN	ELM	LSTM
${\mathrm{DAQ}}_{10}$	85.74	72.66	87.64	85.50	75.15	78.02	**88.35**	87.39
${\mathrm{DAQ}}_{48}$	77.78	68.84	**87.45**	86.99	85.19	80.65	75.81	72.48

**Table 12:** Mean leave-5-day-out cross-validation results (averaged over 10 runs) for the strawberry case-study (%).

Metric	Data	MLP	LR	XGB	RF	SVM	CNN	ELM	LSTM
Accuracy	${\mathrm{DAQ}}_{10}$	76.1	70.9	75.6	73.7	**87.4**	52.0	82.4	67.0
F1	${\mathrm{DAQ}}_{10}$	65.6	70.9	75.6	73.7	**87.4**	52.0	82.4	54.9
Accuracy	${\mathrm{DAQ}}_{48}$	72.4	64.6	72.3	71.3	**75.6**	66.8	61.5	70.4
F1	${\mathrm{DAQ}}_{48}$	70.8	64.6	72.3	71.3	70.4	**75.6**	66.8	57.7

**Table 13:** Mean 10-fold cross-validation accuracy results on data from ${\mathrm{DAQ}}_{10}$, with and without using temperature and humidity information (%).

	MLP	LR	XGB	RF	SVM	CNN	ELM	LSTM
Without	85.74	72.66	87.64	85.50	*75.15*	*78.02*	*88.35*	*87.39*
Features	*87.51*	*76.46*	**89.18**	*86.56*	72.77	n/a	77.28	n/a
Correction	85.13	74.80	87.76	86.45	71.82	73.49	76.58	85.26

**Table 14:** Mean 10-fold cross-validation accuracy results on data from the strawberry case-study (%).

	MLP	LR	XGB	RF	SVM	CNN	ELM	LSTM
Simple	58.75	58.54	55.83	93.67	80.52	76.62	48.94	84.65
Group	47.23	49.09	59.39	72.10	64.58	63.72	47.63	68.01

**Table 15:** Results of MobileNetV3 evaluated on test data batch 2 of the banana ripening case-study.

Test Batch	Image Subset	Accuracy (%)	Recall (%)		Precision (%)	
			Ripe	Overripe	Ripe	Overripe
Batch 2	All data	46.20	41.50	51.92	100.00	99.08
	Daylight	99.53	99.04	100.00	100.00	99.08

**Table 16:** Results of YoloV5 on the strawberry data without confidence thresholding (%).

	Instances	Precision	Recall	mAP50	mAP50-95
All	6183	68.70	41.30	47.70	23.00
Unripe	2772	73.60	43.80	52.20	26.90
Ripe	3411	63.90	38.80	43.20	19.20

**Table 17:** Results of YoloV5 on the strawberry data with confidence = 0.8 (%).

	Instances	Precision	Recall	mAP50	mAP50-95
All	6183	95.90	13.10	54.30	33.90
Unripe	2772	97.00	14.00	55.40	35.00
Ripe	3411	94.70	12.10	53.20	32.70

**Table 18:** Results when binary labels (ripe/unripe) are assigned to the strawberry images.

	All Images	Subset of Well-Lit Images
Number of images	1717	498
Accuracy (%)	26.55%	69.07%
Number of images with detected objects	533	361
Accuracy on images with object found	85.55%	95.29%

**Table 19:** Accuracy of classification on the strawberry dataset where both modalities agree on the class label.

	MLP	CNN	LR
${\mathrm{DAQ}}_{10}$			
Yolov5 and odour model agreed on % of samples (out of 841):	18.79% (158)	17.00% (143)	17.48% (147)
When image and odour agreed accuracy was:	98.73%	99.30%	97.28%
${\mathrm{DAQ}}_{48}$			
Yolov5 and odour model agreed on % of samples (out of 661):	17.25% (114)	17.85% (118)	16.49% (109)
When image and odour agreed accuracy was:	99.12%	97.46%	97.25%

## Data Availability

Data is available upon request.

## Abbreviations

The following abbreviations are used in this manuscript:

CNN	Convolutional Neural Network
CV	Cross-Validation
ELM	Extreme Learning Machine
LDA	Linear Discriminant Analysis
LOBO	Leave-One-Block-Out
LR	Logistic Regression
MLP	Multi-Layer Perceptron
MOX	Metal-oxide
NN	Neural Network
PCA	Principal Component Analysis
PLSR	Partial Least Squares Regression
RF	Random Forest
SVM	Support Vector Machine
